# Cold in the Head

**Published:** 1917-02

**Authors:** 


### "COLD IN THE HEAD"

It has been commonly observed that no immunity is acquired as the result of an attack from a common cold. This, with other attendant peculiarities of coryza and related conditions, has raised the question whether colds can be attributed as largely to the agency of bacteria as has been the tendency during recent years. The very expression "catching cold" is now usually interpreted from the standpoint of infectious disease, and there can be little doubt that many features associated with the genesis of colds suggest the interference of living micro-organisms. Colds spring up suddenly among groups of persons when there are possibilities of a common exposure. Thus an entire family may become afflicted. On the other hand, an acute nasal catarrh or so-called "cold in the head" sometimes promptly follows exposure to drafts in a way that seems to exclude the probability of any special primary infective agent. This diversity of opinion with respect to the etiology of common colds, whereby some writers assert that the latter are always due to bacteria of some sort,[1] while others decline to admit this mode of origin as the only way in which the disturbance in health is created, has given rise to corresponding divergences in the advice as to the hygiene of the subject. The question may be asked, can one become "hardened" to the possibility of attack by suitable living?

To a certain degree at least, resistance to bacterial invasion and immunity to changes in temperature and humidity must be acquired by different physiologic mechanisms. The "Handbook of Therapy," issued by the American Medical Association,[2] takes a middle course in these words:

Whether or not every cold is due to contagion or to a germ, chilling, whether indoors or outdoors, certainly predisposes to colds. It is quite probable that chilling of the surface of the body congests the inner organs and possibly the mucous membranes of the air passages. If the mucous membrane of the nose is congested, it more readily becomes inflamed by irritation or by germs.

With respect to the condition of the nasal respiratory passages, the familiar seat of coryza, Leonard Hill[3] observed that the nasal mucosa became swollen and red in warm, moist air, accompanied by a marked increase in the amount of secretion. On passing from warm to cold air, he found that the mucous membranes became paler, but still remained swollen. He believes that this condition predisposes to disease for the reason that the defensive mechanism of the blood, the immunization properties of the plasma, the cleansing action of the cilia and the phagocytic action of the white blood-cells are all diminished by the cold, while the pathogenic bacteria find a suitable nidus for their growth in the secretion of the swollen mucous membrane. Hill found that keeping the air in motion in warm rooms interfered materially with the hyperemic effect, and believes that in this way the massiveness of direct infection is reduced. He also observed that warm dry air produced less swelling and secretion than warm moist air.

Further facts regarding the exact place of atmospheric changes in the production of respiratory disease have recently been contributed through the scientific work of the New York State Commission on Ventilation.[4] Clinical experiments have been conducted on nearly 150 male subjects including, among others, workers whose occupation in itself was associated with certain distinctive temperatures, such as truck drivers exposed to the varied weather conditions, boiler makers and firemen used to hot, dry atmospheres, and laundry workers who are exposed to heat and humidity. These subjects have been exposed in an experimental chamber at times for several hours to extreme heat and humidity or to cold, and at other times they have been shifted back and forth from one extreme to the other. Ordinarily it was found that heat causes a swelling of the inferior turbinates of the nose, tending to a reduction in the size of the breathing space, and to increase of secretion and reddening of the membranes. The action of cold, as a rule, is just the opposite, namely, a reduction in the size of the inferior turbinates, and a diminution of secretion and color of the membranes. In the industrial workers, mentioned above, whose occupations involved continuous exposure to extremes of heat and cold, these typical changes were not followed. This study was very successful in picturing how the membranes of the nose of such persons are less able to make the adaptations of the normal nose to changes in the atmospheric environment.

A further suggestion that abnormal conditions of a permanent nature may be produced by repeated exposure to overheating was found in observations among long-time workers in hot, moist rooms such as are found in steam laundries. In this group atrophic rhinitis was far more prevalent than among young students. Of course, contagion may be an added etiologic factor under such circumstances. Moist heat appears to produce greater changes than does dry heat in the nasal passages. There is some physiologic indication that the reaction of the nasal structures to atmospheric changes is primarily direct and local, rather than reflex; but the evidence on this point is by no means conclusive.

Cocks, who conducted most of these investigations on the respiratory tract, points out that turbinate responses are very delicate and that the changes observed are by no means always constant. Obviously it is important to place the subject of "colds in the head" on a more scientific basis as the result of investigations such as those here outlined, which do not feature the problem of pathogenesis solely from the bacteriologic standpoint. The reactions in the nasal mucous membranes produced by changes in atmospheric environment are, says Cocks, too frequent and too definite to be disregarded. Accordingly, he is convinced that the theory of bacterial infection as the sole cause of catarrhal inflammations of the upper air passages is not tenable, since the changes produced by environment must materially affect the incidence of infection.

Elsewhere[5] in this issue, page 1180, Foster reports experiments from which he concludes that common colds—of a certain type at least—are infectious and that the causative virus occurs in nasal secretions. The Berkefeld filtrates of subcultures of this virus he found were capable of producing symptoms of an acute cold in healthy persons. His results are significant and should be followed by experiments for further evidence pointing toward their confirmation.—*Journal A. M. A.*, April 15, 1916.

---

[1] Compare Chodunsky: Erkältung und Erkältungskrankheiten, Vienna, 1907.

[2] Osborne, O. T.; Fishbein, Morris, and Salisbury, J. H.: Handbook of Therapy, Chicago, 1915, p. 206.

[3] Hill, Leonard, and Muecke, F. B.: Colds in the Head, Lancet, London, May 10, 1913.

[4] The newest data are reported in the outline of the activities of the commission, presented by the chief of the investigating staff, George T. Palmer, at the annual meeting of the American Society of Heating and Ventilating Engineers, New York, January 20, 1916. See also Cocks, G. H.: Experimental Studies of the Effect of Various Atmospheric Conditions upon the Upper Respiratory Tract, read before the American Laryngological Rhinological and Otological Society, at the Twenty-first Annual Meeting, in Chicago, June 15-16, 1915.

[5] Foster, G. B.: The Etiology of Common Colds, THE JOURNAL A. M. A., this issue, p. 1180.

